# Isomeric Mono-, Di-, and Tri-Bromobenzo-1H-Triazoles as Inhibitors of Human Protein Kinase CK2α

**DOI:** 10.1371/journal.pone.0048898

**Published:** 2012-11-14

**Authors:** Romualda Wąsik, Patrycja Wińska, Jarosław Poznański, David Shugar

**Affiliations:** 1 Institute of Biochemistry and Biophysics PAS, Warszawa, Poland; 2 Faculty of Chemistry, Warsaw University of Technology, Warsaw, Poland; 3 Division of Biophysics, Institute of Experimental Physics, University of Warsaw, Warszawa, Poland; National Cancer Institute at Frederick, United States of America

## Abstract

To further clarify the role of the individual bromine atoms of 4,5,6,7-tetrabromotriazole (TBBt), a relatively selective inhibitor of protein kinase CK2, we have examined the inhibition (IC_50_) of human CK2α by the two mono-, the four di-, and the two tri- bromobenzotriazoles relative to that of TBBt. Halogenation of the central vicinal C(5)/C(6) atoms proved to be a key factor in enhancing inhibitory activity, in that 5,6-di-Br_2_Bt and 4,5,6-Br_3_Bt were almost as effective inhibitors as TBBt, notwithstanding their marked differences in pK_a_ for dissociation of the triazole proton. The decrease in pK_a_ on halogenation of the peripheral C(4)/C(7) atoms virtually nullifies the gain due to hydrophobic interactions, and does not lead to a decrease in IC_50_. Molecular modeling of structures of complexes of the ligands with the enzyme, as well as QSAR analysis, pointed to a balance of hydrophobic and electrostatic interactions as a discriminator of inhibitory activity. The role of halogen bonding remains debatable, as originally noted for the crystal structure of TBBt with CK2α (pdb1j91). Finally we direct attention to the promising applicability of our series of well-defined halogenated benzotriazoles to studies on inhibition of kinases other than CK2.

## Introduction

Protein kinase CK2, a Ser/Thr kinase (also known to phosphorylate Tyr residues), the most pleiotropic of all protein kinases, plays a key role in cell growth, differentiation, cell death and survival, and is a highly potent suppressor of apoptosis. It has been reported to be dysregulated and overexpressed in all cancers hitherto examined, and has long been considered a key target for cancer chemotherapy [1], underlining the importance of development of low-molecular weight selective inhibitors of this enzyme, as well as its two catalytically active subunits CK2α and CK2α’.

The first reported low-molecular weight inhibitors of this enzyme, 4,5,6,7-tetrabromobenzotriazole (TBBt, also known as TBB) [2] and 4,5,6,7-tetrabromobenzimidazole (TBBz) [3], both shown to be cell-permeable [4], exhibit K_i_ values in the low µM and sub-µM range, and were found to be relatively selective when tested against a panel of more than 60 other kinases [5]. Both were subsequently found to be precursors of more potent inhibitors, analogues with various substituents on the triazole or imidazole rings, some with K_i_ values in the nM range, reviewed, amongst others, by Zien et al. [6] and Battistutta et al. [7].

Notwithstanding the high structural similarity between TBBt and TBBz, they differ significantly in their mode of binding to CK2a, with a root mean square deviation (RMSD) of over 2.5 Å between corresponding locations of the Br atoms within the binding pocket. The complex with TBBz is stabilized by two well-defined halogen bonds [7], and an analogous pattern of two halogen bonds involving the same aminoacid residues, but making short contacts with other bromine atoms of the ligand, observed in complex with 3,4,5,6,7-pentabromo-1H-indazole [8]. No such bonds were observed in the structure of the complex with TBBt [9]. However, in the latter manuscript, the authors inadvertently overlooked a short Br…Nε(Arg47) contact (2.99 Å), further discussed below (see Discussion).

The foregoing stimulated development of many other, structurally unrelated, potent selective inhibitors, culminating in the appearance of Cylene’s oral CX-4945, the first low-molecular weight CK2 inhibitor to reach the clinic in phase I and phase II clinical trials, in patients with solid tumors, multiple myeloma, and Castleman’s disease [10].

The biological importance of the halogeno benzotriazoles and benzimidazoles is further underlined by the fact that they are selective inhibitors of various protein kinases [8], [11], [12]. Moreover, some of them efficiently inhibit the NTPase/helicase activities of hepatitis C and related viruses [13]. In addition, Townsend and coworkers have demonstrated that a number of halogeno benzimidazole nucleosides are potent inhibitors of some herpesviruses, one of which is presently in clinical trials for HCMV infections [14].

The foregoing reflects the current widespread interest in elucidating the role of halogenated ligands in biological systems, extensively reviewed, amongst others, by Aufinger et al. [15], Voth & Ho [16], Parisini et al. [17], Grant & Lunney [18], Lu et al. [19] and Rendine et al. [20].

We have previously demonstrated [21] that replacement of one of the bromines of TBBt, that at C(5), by a variety of other substituents, differing in size, electronegativity and hydrophobicity, resulted in significant changes in ionic equilibrium, protomeric preference for the neutral form, and inhibitory activity against CK2α. In general, the hydrophobicity of the anionic form of the ligand was found the principle factor influencing its inhibitory activity.

**Figure 1:**
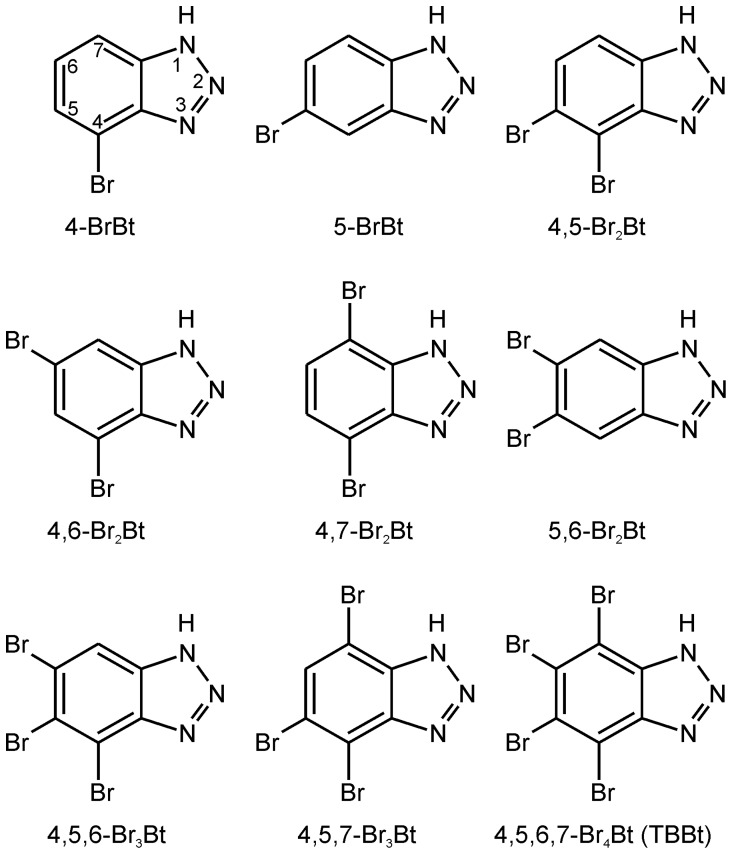
Structures of all possible halogenated derivatives of benzotriazole.

To further define the role of the individual Br atoms of TBBt, as regards potency and selectivity as inhibitors of CK2α, we have synthesized all the possible two mono-, four di-, and two tri- bromobenzotriazoles [22] (Figure 1), and herein we model the structure of their complexes with CK2α and examine the relation between their physico-chemical properties and inhibitory activities *vs.* CK2α.

## Results

### Inhibitory Activities – IC_50_

The IC_50_ values for inhibition of CK2α by all the bromobenzotriazole derivatives are shown in Table 1, and the dose-response curves are presented in Figure S1. The parent Bt was found totally inactive, but all the halogenated derivatives were active, with inhibitory activity dependent both on the number of bromine atoms attached to the benzene ring, as well as on their location. Generally, substitution on the central vicinal C(5)/C(6) carbons decreases IC_50_ much more than that on the peripheral C(4)/C(7) carbons. Thus 5-BrBt, 5,6-Br_2_Bt and 4,5,6-Br_3_Bt are the most active mono-, di- and tri-brominated benzotriazoles, respectively. Moreover the inhibitory activities of 5,6-Br_2_Bt, 4,5,6-Br_3_Bt and TBBt are almost comparable, suggesting that bromination of both C(5) and C(6) carbons is crucial for efficient interaction of halogenated Bt derivatives with CK2α.

**Table 1:** Experimental values for triazole proton dissociation (pK_a_), aqueous solubility (C_w_), and inhibitory activity (IC_50_) against CK2α compared with molecular volumes of brominated Bt derivatives, *ab initio* derived free energies of proton dissociation (ΔG_diss_), free energies of solvation of the anionic forms, free energy of binding to CK2α estimated with the aid of Autodock for ligands both in neutral and monoanionic state (ΔG_bind_), average ligand movement upon 3 ns Molecular Dynamics of the complex with CK2α in aqueous solution (RMFS), and the displacement between average ligand location estimated from 3 ns MD simulations in aqueous medium from the location of TBBt in the crystal structure with CK2α (RMSD).

Ligand	pK_a_ a	C_w_ a [M]	IC_50_ b [µM]	V_mol_ c [Å^3^]	ΔG_diss_ c [kcal/mol]	ΔG_solv_ c [kcal/mol]	ΔG_bind_ b	[kcal/mol]	RMSF^b^ [Å]	RMSD^b^ [Å]
							neutral	anion		
**Bt**	8.56	1.96·10^−1^	>2000	127.1	59.5	−59.9	6.3	5.7	0.47	2.99
**4-BrBt**	7.08	3.87·10^−3^	119±10	151.1	56.7	−53.3	7.0	6.5	0.47	0.76
**5-BrBt**	7.55	2.77·10^−3^	26±3	151.3	58.1	−50.7	6.8	6.5	0.55	0.57
**4,5-Br_2_Bt**	6.49	4.99·10^−4^	10.6±0.9	172.1	54.3	−46.8	7.9	7.1	0.42	0.26
**4,6-Br_2_Bt**	6.38	2.34·10^−4^	10.0±2.2	174.7	54.2	−45.5	7.5	7.1	0.60	0.76
**4,7-Br_2_Bt**	5.84	1.98·10^−3^	72±11	174.9	53.4	−48.0	7.2	6.9	0.40	0.81
**5,6-Br_2_Bt**	6.93	2.31·10^−4^	0.56±0.02	172.4	55.8	−44.4	7.7	7.1	0.46	0.87
**4,5,6-Br_3_Bt**	5.91	6.23·10^−5^	0.38±0.02	192.8	52.3	−40.9	8.6	7.7	0.45	0.13
**4,5,7-Br_3_Bt**	5.38	1.04·10^−3^	5.8±0.9	195.9	52.2	−41.9	8.1	7.8	0.46	0.64
**TBBt**	4.78	2.18·10^−4^	0.27±0.07	213.0	49.7	−38.0	8.9	8.4	0.52	0.71

a Experimental data determined for all compounds under identical conditions [21], [22].

b This work.

c Values calculated previously using the same approach for all compounds [21], [22].

### QSAR Analysis of Inhibitory Activities

**Figure 2:**
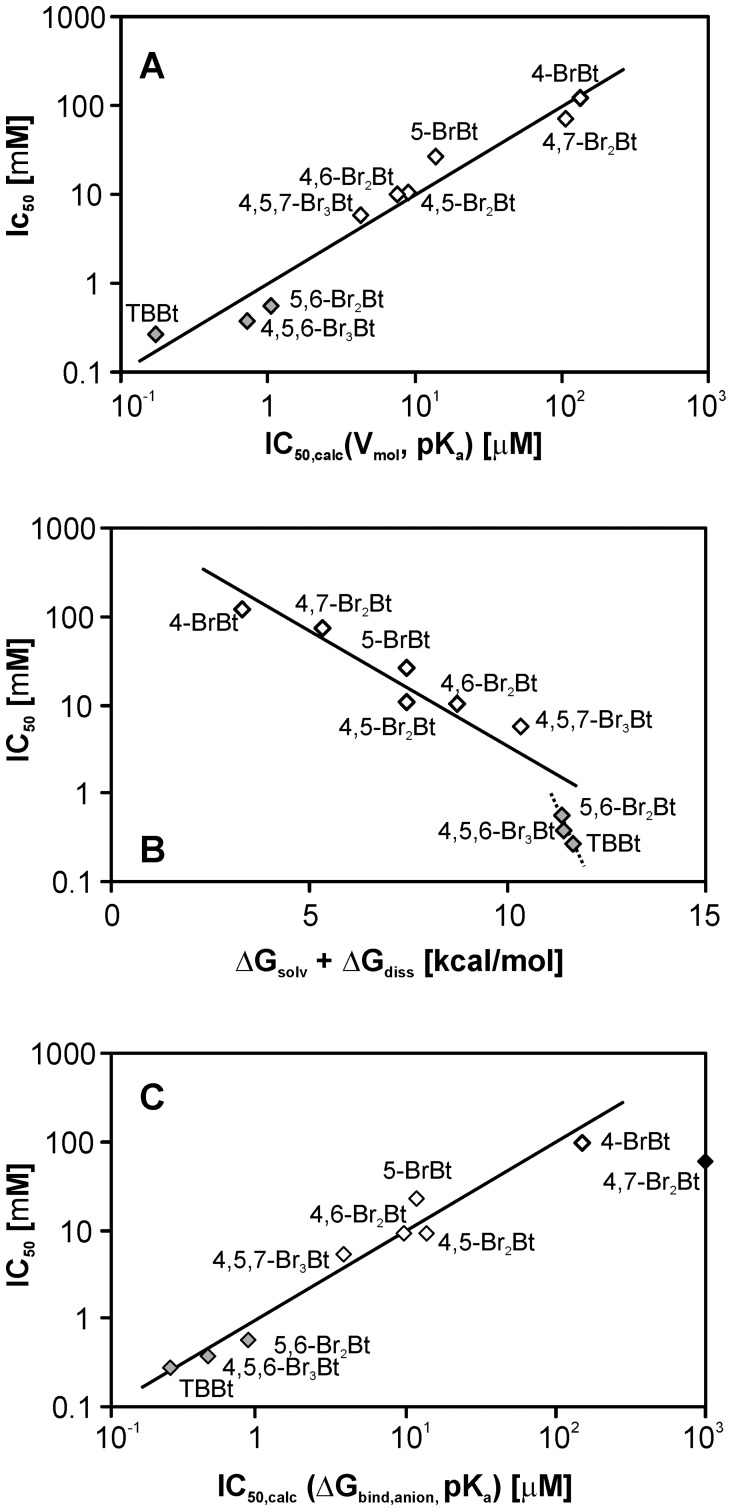
Inhibitory activities (IC_50_) of brominated Bt derivatives predicted on the basis of: (A) V_mol_ and experimental pK_a_; (B) *ab initio* derived ΔG_solv_(anion) and ΔG_diss_ free energies; and (C) autodock-derived free energy of binding (ΔG_bind_). All relations point to predominance of hydrophobic interactions (V_mol_ or ΔG_solv_(anion)), accompanied by protonation of the anionic form of the ligand upon binding to CK2α (pK_a_ or ΔG_diss_) (see text for details).

Principle Component Analysis points to two basic parameters of the halogenated ligands (V_mol_, pK_a_) as the major determinants of inhibitory activity, 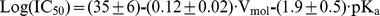
_,_ R^2^ = 0.92, F(2,7) = 37.7 (R^2^ = 0.95, F(2,6) = 68.5 when Bt is excluded from the analysis, see Figure 2A for details). According to the principles of Lum-Chandler-Weeks’ theory of hydrophobic solvation (LCW) [23], the molecular volume of a solute molecule is a direct measure of the free energy of non-specific hydrophobic hydration, and the pK_a_ contribution clearly indicates that the ionic state of a possible anionic form of a ligand in solution changes upon binding to CK2α, putatively favoring its neutral form. In general, for inhibitors of similar size, the higher the pK_a_, (i.e. the less dissociated at neutral pH), the stronger the binding to CK2α and, consequently, the lower the expected IC_50_ value.

But the most effective predictor of inhibitory activity is based on the sum of QM-derived free energies of proton dissociation, ΔG_diss_, and solvation of the anionic ligand form, ΔG_solv_(anion), 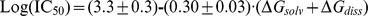, R^2^ = 0.94, F(1,8) = 116 (Figure 2B).

Hence, again, binding to the enzyme is largely driven by hydrophobic interactions with the monoanionic form of the ligand, as represented by ΔG_solv_(anion), which then becomes preferably neutral on binding in the enzyme pocket (factor ΔG_diss_). The small, but significant, deviations observed for 5,6-Br_2_Bt, 4,5,6-Br_3_Bt and TBBt suggest that, for these three derivatives, another pattern of interactions directs complex formation. Further analysis of the modeled complexes demonstrated that the two bromine atoms at C(5) and C(6) are virtually involved in a network of halogen bond-like interactions with the proximal carbonyl group of Val116, and an aromatic nitrogen of the His160 side-chain and the guanidine group of Arg47. Molecular Dynamics (MD) screening demonstrated that, in the case of 4,5,7-Br_3_Bt, an additional water molecule is directly involved in binding of the ligand, thus compensating for the absence of the large Br atom at C(5) or C(6) which, in the crystal structure of TBBt with CK2α, was originally located in the proximity of Asn118 and His160 [9].

### Molecular Modeling

Binding of all analogues to CK2α was analyzed by mining the free energy of binding, estimated by ligand docking to the protein, the location of which was restricted to the region proximal to the TBBt binding site.

Estimated values of free energy of binding (Table 1) are well correlated with inhibitory activities (Figure 2C), including the parent Bt (outside the diagram). For 5-BrBt and 5,6-Br_2_Bt, binding was underestimated by approximately 0.8 kcal/mol. However, the docking procedure was performed in the absence of explicit water molecules, to avoid the almost insurmountable problem of simultaneous docking of three independent molecules, namely – a given ligand and the two water molecules located in the binding pocket in the complex of CK2α with TBBt. But the absence of explicit water molecules, directly involved in ligand binding, causes systematic displacement of ligands toward Lys68 and Glu81.

**Figure 3:**
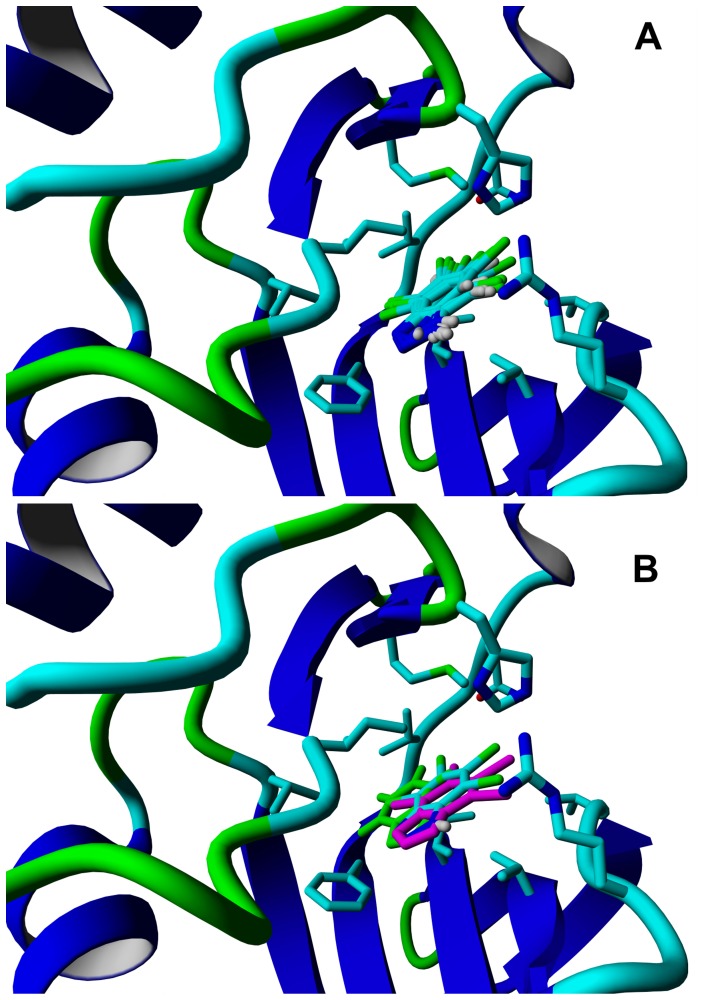
Location of all nine halogenated Bt derivatives in complex with CK2α. For each ligand the average location determined from the 3 ns trace of Molecular Dynamics performed in the presence of explicit aqueous solvent is presented in relation to X-ray structure of CK2α (pdbj91). All ligands were found to bind in the same orientation (see panel A), in the position almost identical to that found for TBBt in the crystal structure of the complex with CK2α (see panel B, TBBt, from PDB, in magenta and putative location of Bt in green). The lowest-energy structures identified in 3 ns MD traces are presented in Figure S3.

To further clarify this problem, an additional 5 ns molecular dynamics was performed for each ligand in the presence of explicit water molecules. Analysis of the last 3 ns of each MD trace indicated that, for all ligands, their flexibility inside the CK2α binding pocket is comparable, and the root mean square fluctuation (RMSF) values, which are a measure of differences in ligand locations in snapshots of the MD trajectory, vary over a narrow range of 0.4–0.6 Å. It follows that the binding pocket may readily adapt to the smaller ligands, and, consequently, the entropic contribution arising from ligand translational degrees of freedom is almost the same for all the ligands. For all nine brominated Bt derivatives the average location was found almost the same (Figure 3A), and identical to that of TBBt in the crystal structure with CK2α (RMSD in the range 0.4–0.8 Å) (Figure 3B). The only significant deviation was noted for the parent Bt (3.0 Å), which was found inactive.

The foregoing shows that all the halogenated Bt derivatives bind in the same orientation to CK2α. The correlation between experimental IC_50_ values and the energy terms, estimated both from the docking procedure (Figure 2C, semi-empirical parametrization of the “classical” forcefield describing Van der Waals, electrostatic, H-bonding interactions, and desolvation), and directly from *ab initio* QM calculations for solvation (Figure 2B), further demonstrate that the putative contribution of halogen bonding is significantly smaller than the other energy terms used in the calculations, if at all. In fact, detailed inspection of several conformational degrees of freedom for the protein showed that possible halogen bond-promoting conformations are not realized in the CK2α -TBBt complex, as illustrated by the His160 residue, for which a flip of the aromatic ring would have enabled interaction of Nε2 with a central (C5/C6) halogen atom. The only halogen-bond promoting conformation was found for the π-Br interaction involving the Nε of Arg47 and a peripheral (C4/C7) halogen atom of TBBt, as also found for one of the two protein molecules located in the crystal asymmetric unit [9].

## Discussion

**Figure 4:**
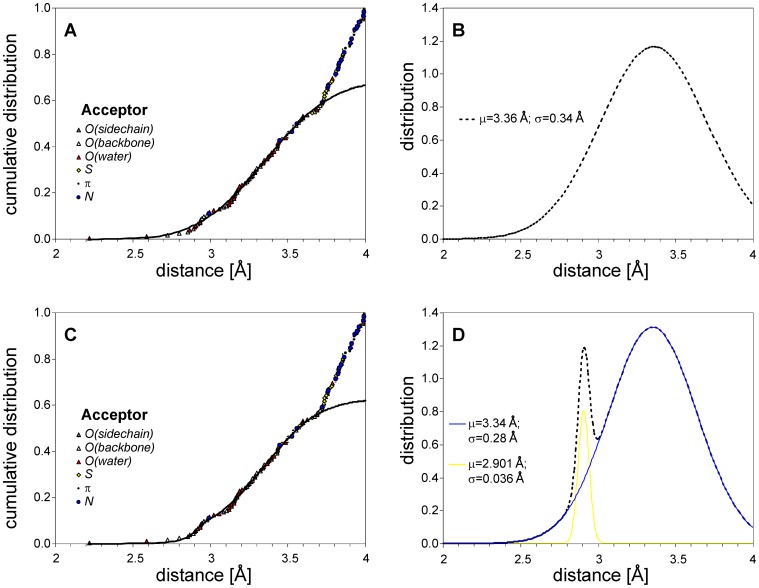
Distribution of short halogen-acceptor (O, N, S, π system) contacts identified in 21 accessible structures of complexes of CK2α with halogenated ligands. The Gaussian cumulative distribution was fitted to the crystallographic data for halogen to donor distance (solid line in panel A) and, according to the Anderson-Darling test, experimental data up to 3.7 Å agrees with a normal distribution (panel B). However, pairs separated by more than 3.7 Å are overrepresented, clearly limiting the maximal distance for eventual halogen-bonding interactions to the sum of donor and acceptor VdW radii. Note that an isolated water molecule (red triangles in panels A, C) is an equally favorable acceptor to the protein (O, N, S, π-electrons). The cumulative distribution of the experimental data is visibly better represented by a bi-normal distribution (panel C), in which the contribution of an additional narrow peak represents putative halogen-bonding (panel D). This is additionally supported by the distribution of angles X…Acc-C and C-X…Acc, which, for short halogen-acceptor distances, are substantially restricted to the regions favoring halogen bond formation (see also Figure S2).

The propensity of the CK2α binding pocket to bind a halogenated ligand must be considered in terms of the topology of potential halogen-bond acceptors and the internal flexibility of the protein. We have performed a detailed analysis of all of 21 accessible structures of complexes of CK2 with ligands carrying at least one halogen atom (X = Cl, Br or I, see Table S1). All of them were inspected using a 4 Å threshold, to identify the distribution of distances between halogen atoms and proximal halogen bond acceptors (i.e. backbone carbonyl oxygens, side-chain oxygens, nitrogen and sulphur atoms, and oxygens of water molecules). The resulting distribution of Acc…X distances (analyzed in a cumulative manner to prevent the necessity of clusterization) revealed the existence of a broad local maximum corresponding to a normal distribution with a mean value of 3.34 Å and a standard deviation of 0.28 Å (Figure 4). The substantial break observed for longer distances strictly corresponds to the sum of the VdW radii of dominant bromine and oxygen atom pairs (3.7 Å), thus clearly setting the upper limit for eventual halogen bonding. It should also be emphasized that the normal distribution is somewhat perturbed, displaying a visible shoulder centered at ∼3.0 Å. Although, according to the Anderson-Darling test, the assumption of normality of the observed distance distribution could not be rejected, superposition with an additional normal distribution visibly improves the agreement between the model and the observed data. The additional extremely narrow distribution (mean 2.90 Å, standard deviation 0.04 Å) confirms overrepresentation of some types of donor-acceptor pairs. Interestingly, the potential donors of a strong halogen bond, with the sole exception of the Arg47 side-chain nitrogen engaged in the complex with TBBT (1j91), are oxygen atoms of either a backbone carbonyl or an isolated water molecule. Amongst theese, the preferred topology of C-X…Acc-C remains acceptor-specific, significantly differentiating between carbonyl and hydroxyl acceptors (see Figure S2). Such a strong geometric preference for these interatomic interactions denotes them as halogen bonds [15], while the accompanying broad distribution describes a rather highly non-specific balance of VdW and electrostatic interactions. It should further be noted that the distribution of donor-acceptor distances remains identical for protein carbonyl and water hydroxyl oxygen atoms (Figure 4).

Of the 21 structures listed in Table S1, 18 exhibited X…Acc contacts shorter than 3.7 Å, considered, according to the actual paradigm, to be halogen bonds. Amongst these, the most abundant halogen bond acceptors are the carbonyl groups of Glu114 and Val116, both of which account for 80% of identified halogen bonds. The mean geometry of these bonds, i.e. X…O distance, and C-O…X and O…X-C angles, demonstrate that the spatial organization of the CK2α binding pocket clearly prefers the Val116 carbonyl as the halogen-bond acceptor (average length 3.01±0.19 Å, and angles C = O…X = 131.5±3.4°, O…X-C = 170.8±7.0°) over Glu114 (3.31±0.16 Å, 162.3±5.5°, 145.4±4.4°, respectively, see Table S1 for details). Three halogen bonds, identified in structures 1J91, 2PVK and 3NGA, are in accord with the concept of orthogonal halogen bonds proposed by Voth et al. [24].

It should again be emphasized that TBBt in complex with CK2α (1J91) does not exhibit any Br…O halogen bonds (although the distance 4.0 Å between the Val45 carbonyl oxygen and Br13 of TBBt might be regarded as halogen bond-promoting), whereas its close structural analogue, tetrabromobenzimidazole, forms two halogen bonds (see 2OXY in Table S1). This indicates that the free energy of electrostatic and hydrogen-bonding interactions of the triazole ring of TBBt with a proximal water molecule, and the charged side-chains of Lys68 and Asp175, exceed the gain of eventual formation of two halogen bonds with the carbonyl groups of Glu114 and Val116, possible in the alternative TBBz-like location of the ligand. This results in significant differences in location of both ligands in the CK2α binding pocket, notwithstanding that the molecules are of the same size, differing only by replacement of the N(2) nitrogen in TBBt by a carbon in tetrabromobenzimidazole.

Spatial organization of a majority of the low-energy structures of complexes of brominated benzotriazoles with CK2α, derived from our docking procedure, and resembling that of the TBBt complex in the X-ray structure (1J91), also point to predominance of electrostatic interactions over halogen bonding. As suggested by Battistutta et al. [7], halogenated benzotriazoles, especially those carrying a negative charge distributed on the triazole ring, should bind in a manner similar to TBBt.

The consensus residues that participate in binding of all ligands in over 80% of low- energy structures are Val53, Ile66, Lys68, Val95, Phe113, Val116, Ile174 and Asp175, generally in accord with the CK2α binding site identified by Battistutta et al. [25]. With our mono- di-, tri- and tetra-bromo benzotriazoles, additional consensus residues that make contacts in more than 50% of the structures are Glu114, His160, Met163 and Trp176. This pattern of interactions is in accord with our previous simulations for a series of symmetrically substituted Bt [26] and 5-substituted derivatives of Bt and 4,6,7-Br_3_Bt [21], and is also consistent with the interaction scheme proposed by Battistutta et al. [7]_,_ with the exception of Val45, shown to make contacts preferentially with ligands bound in an orientation analogous to that of TBBz, but not that of TBBt.

The order of importance of interactions controlling ligand activity (hydrophobic, VdW, electrostatic) [7] agrees with our experimental IC_50_ data, as well as with the energy terms derived from molecular modeling.

**Figure 5:**
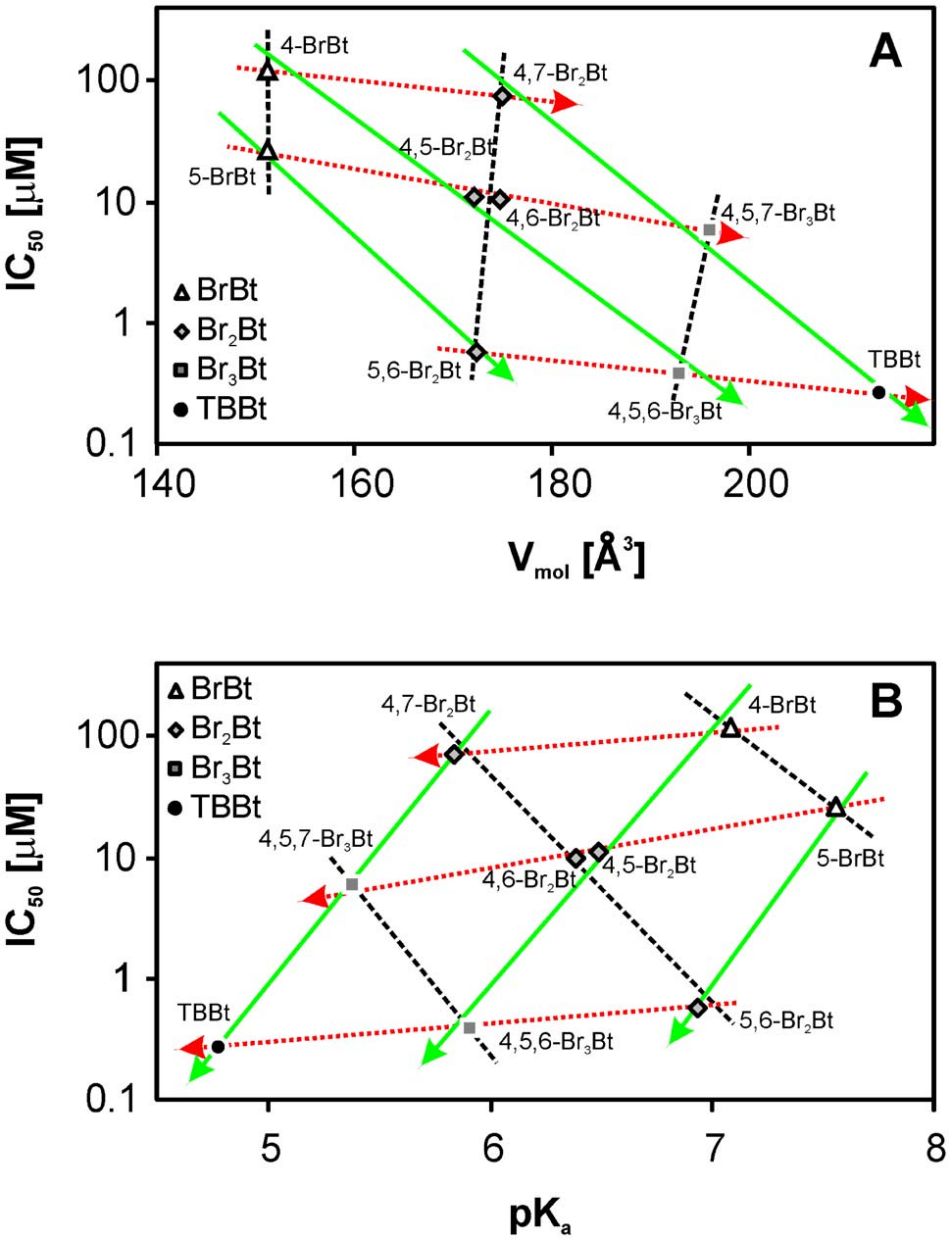
Schematic representation of the effects of stepwise bromination of the benzene ring of benzotriazole (Bt), including interdependence of the molecular volume (A) and pK_a_ (B) of the products with their IC_50_ for inhibition of protein kinase CK2α: (a) Green arrows follow sequential bromination of the central vicinal C(5)/C(6) atoms, leading to a moderate decrease of pK_a_ and a large decrease in IC_50_; (b) Red arrows illustrate the lesser effects of bromination of the peripheral C(4)/C(7) atoms, resulting in a significant decrease of pK_a_, with virtually no gain in inhibitory activity; (c) Black lines link products with the same number of bromine atoms, but substantially differing in pK_a_ and IC_50_, culminating in the di- and tri-brominated derivatives with inhibitory activities comparable to that of TBBt. Note that the parent Bt (pKa 8.56), which is not an inhibitor (IC_50_>2 mM), is located outside the diagrams.

Our overall results point to hydrophobic interactions as the main force driving ligand-CK2α interactions, but electrostatic contributions also appear important. Thus, for isomers carrying the same number of halogen atoms on the benzene ring, the inhibitory activity depends on the location of bromination sites (Figure 5A, black dashed lines). Moreover, a general correlation is observed between pK_a_ for dissociation of the triazole proton and inhibitory activity (Figure 5B, pattern of black dashed lines connecting data for compounds of the same size). Evidently, the less dissociated forms (displaying higher pK_a_ values) are the most active within the series. The almost horizontal lines, displaying the trend of the data for residues carrying the same number of Br atoms attached to C(5)/C(6), but differing by the number of Br atoms attached to C(4)/C(7), demonstrates that the enhanced inhibitory effect of an increase in hydrophobicity of the benzene ring is almost nullified by a decrease of the pK_a_ for dissociation of the triazole proton (Figure 5, red arrows). By contrast, bromination on the vicinal C(5)/C(6) visibly increases inhibitory activity (Figure 5, green arrows).

All studied ligands display high structural similarity, and their locations in the CK2α pocket remain almost identical. Hence competitive inhibition, analogous to thast of TBBt, may be expected for all halogenated benzotriazoles. Consequently, for a given concentration of protein used in enzymological tests, enzymatic activity is proportional to the relative population of ligand-free protein, which is approximately proportional to K_d_. Ligand binding to the enzyme is generally driven by the difference in free energies of bound and unbound states. Consequently, the ionic equilibrium of the free ligand in solution influences the free energy of binding. The evident contribution of the ionic form of an inhibitor in aqueous medium at neutral pH (Figure 5B, see also QSAR approach in Figure 2) shows that the anionic ligand becomes neutral upon binding to CK2α. This is further supported by analysis of the lengths of nitrogen-nitrogen bonds in the triazole ring of TBBt in complex with CK2α (pdb1j91) [9], which are definitely not equal (1.242 and 1.419 Å). This, in turn, confirms the pattern of double and single N-N bonds characteristic for the neutral benzotriazole with a proton attached to either N(1) or N(3), in accordance with solution NMR data [21], [22]. In this view, the pK_a_ for proton dissociation in aqueous medium becomes a major factor affecting binding, and inhibitory activity.

On the other hand, TBBz is 4-fold less active than TBBt [5], pointing to the importance of the negatively charged triazole ring for efficient binding to CK2α. In this view, our finding that 5,6-Br_2_Bt is the most active dibromo isomer points to the role of hydrogen bonding properties of the triazole ring itself rather than the effect of the formal negative charge located on it.

The foregoing is also supported by the moderate activity of 4,5,6,7-tetrachloro-benzotriazole, which is less active than TBBt [3], because substitution of bromine by chlorine decreases the hydrophobicity, but does not significantly change the pK_a_ [26], and the low, but detectable, activity of 4,5,6,7-tetramethyl-benzotriazole [3], which is much less polar, and in the neutral form at physiological pH [26].

It may be concluded that the balance of hydrophobic and electrostatic interactions are the main forces driving the binding of brominated benzotriazoles to CK2α. The dominant effect of permutation of bromination sites simply suggests that a decrease in halogenation of known multiple halogenated inhibitors may result in significant enhancement of their activity.

However, it should be noted that enzymatic dehalogenation may possibly occur *in vivo*, as demonstrated for reductive dehalogenation of polyhalogenated phenols [27], [28] and haloalkanes [29] by bacteria, or iodotyrosine metabolism in mammals [30].

Structural studies of CK2α-ligand complexes show numerous hydrophobic contacts, demonstrating that hydrophobic inhibitors are favored. Moreover, unfavorable hydrophobic solvation moves the protein-ligand equilibrium towards the bound state. But the increase in drug hydrophobicity is limited by the minimal solubility required for drug administration. For TBBt, which is very poorly soluble in its neutral form, addition of DMSO is required, even for biochemical studies, in which a final 2% DMSO concentration was used (not affecting enzyme activity). Solubilities of two identified good inhibitors, 5,6-Br_2_Bt and 4,5,6-Br_3_Bt, were found to be as low as for TBBt at neutral pH, and visibly higher in acidic solution. However, there are alternative approaches for administration of such hydrophobic compounds, based on formation of water-soluble supramolecular complexes of drugs with carrier molecules. These include cyclodextrins of an appropriate size [31] or some calix- [4]-arenes [32]. It should also be noted that 5,6-Br_2_Bt is almost neutral at physiological pH, which may eventually result in a significant decrease of the undesired side effect of ribosome depolarization observed for TBBt [33], which is anionic in physiological conditions.

Finally, we direct attention to the fact that there are numerous reports on inhibition of various protein kinases by halogenated benzotriazoles and benzimidazoles, and their nucleosides [12], [34], for some of which the site(s) of halogenation were not unequivocally identified [11]. The present series of well-defined halogeno benzotriazoles should prove useful in more comprehensive studies on inhibition of kinases other than CK2, and suggest, furthermore, that it would be desirable to synthesize the corresponding series of halogeno benzimidazoles.

## Materials and Methods

### Chemicals

[γ-^32^P] ATP (3000 Ci/mmol) was obtained from Hartmann Analytic GmbH. P81 (2.3 cm) filters were from Whatman. The CK2 synthetic peptide substrate (RRRDDDSDDD) was purchased from Biaffin GmbH & Co KG. CK2α was from KinaseDetect Aps. All reagents were of analytical grade. Synthetic brominated ligands were prepared according to previously reported procedures [21], [22].

### Assays of CK2α Activity and Inhibition Studies

CK2α activity was monitored using the P81 filter isotopic assay [35]. The reaction mixture contained 20 mM Tris-HCl, pH 7.5, 20 mM MgCl_2_, 20 µM DTT, 20 µM peptide substrate, 0.5 mM β-glycerol, 0.1 mM EGTA, 10 µM ATP (200–300 cpm/pmol) and CK2α (0.4 µg/µl). The reaction was initiated with enzyme in a total volume of 50 µl, and incubated at 30°C for 20 min. 10 µl of a reaction mixture was spotted onto a P81 filter. The filter papers were washed 3× with 0.6% phosphoric acid, once with 95% ethanol, then counted in a scintillation counter (Canberra-Packard).

Inhibition of the enzyme was determined by following the decreases in enzyme activity with a minimum of 7 concentrations of each inhibitor (from a stock solution in DMSO) in the range 0.016–250 µM. This led to introducition of 4% DMSO in the incubation mixtures, which was determined to have no effect on enzyme activity. IC_50_ values were then estimated with the use of the GOSA-fit (Global Optimization by Simulated Annealing) Bio-Log program according to the equation:
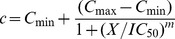
where c is the number of counts for a given concentration of the ligand (X), IC_50_ is the concentration of inhibitor that decreases the rate of phosphorylation by 50% at a fixed concentration of [γ-^32^P] ATP, and C_max_ and C_min_ are the asymptotic values.

### Theoretical Methods

Docking was performed with the aid of the Autodock program [36] implemented in the Yasara-model package [37]. Interactions between ligands and the protein were scored by Van der Waals, electrostatic, hydrogen bonding and desolvation energy terms adopted from the Amber03 force field. The docking procedure for location of ligands was performed in a space restricted to 5 Å from the reference location of TBBt in its crystal structure with CK2α (PDB1J91). Taking into account the N(1)-H ↔ N(3)-H protomeric equilibrium for neutral asymmetric molecules, all 16 possible permutations of Br->H substitutions were analyzed. The initial structure of the protein was taken from the crystal structure of its complex with TBBt, while the topology and point charges for the ligands were adopted from *ab initio* calculations. For each ligand, a total of 999 rounds of restricted random docking was performed, and the ensemble of estimated ligand locations was then clustered according to an assumed 3 Å threshold, found sufficient to distinguish between the two possible proton locations in the symmetrical ligand. The lowest energy-cluster was taken as the structural representation of the complex.

Molecular Dynamics analysis was carried out with the aid of the Yasara-Model [37], using the standard Yasara2 force field [38], further extended for all ligands by adding *ab initio* derived topologies and charge distributions. Starting structures of complexes with various ligands were adopted from the crystal structure of CK2α complex with TBBt (PDB1J91). All water molecules closer than 6 Å from the protein in the original PDB structure were preserved, and then a cube with dimensions 78×66×59 Å was filled with additional water molecules to give an average solvent density of 1.004 g/ml. Coordinates of protein residues distal by more than 6 Å from the initial ligand location were fixed. Similarly all distal water molecules (threshold 8 Å) were fixed. For each of the 16 possible permutations of Br → H replacements, 5 ns MD simulations were performed, and the last 3 ns subjected to more detailed analysis.

All the numerical models, including iterative QSAR reduction by means of the lowest eigenvalue vector, were performed using the Marquand algorithm [39] implemented in the GnuPlot program [40].

## Supporting Information

Figure S1
**Determination of inhibitory activity of the benzotriazole derivatives studied.** The plots were obtained using Graph Pad Prism by fitting the experimental data (minimum three independent experiments for each of the tested compound) to the model: 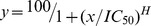.(TIF)

Figure S2 **C-X…Acc (A) and X…Acc-C (B) angles determined for halogen bonds identified in 18 X-ray structures of CK2α, represented as a function of X…Acc distance.** The region of short halogen-acceptor contacts (shadowed rectangle) shows visibly restricted values of both angles. Note that water molecule (red triangles in panel A) differs in optimal geometry from protein acceptors (empty triangles). A newly identified perpendicular halogen bond between TBBt and Arg47 is marked in blue.
(TIF)

Figure S3 **Lowest energy structures of benzotriazole and its Brominated derivatives in complex with human CK2α.**
(TIF)

Table S1 **Short contact between halogen atom and potential halogen bond acceptors identified in 18 of 21 complexes of CK2α with halogenated ligands, accessible in the Protein Data Bank.**
(DOC)
