# Supplementary figures and images for: Isomeric Mono-, Di-, and Tri-Bromobenzo-1H-Triazoles as Inhibitors of Human Protein Kinase CK2α
Source: PLoS One. 2012 Nov 14;7(11):e48898. doi: 10.1371/journal.pone.0048898 (PMC3498355; doi:10.1371/journal.pone.0048898)

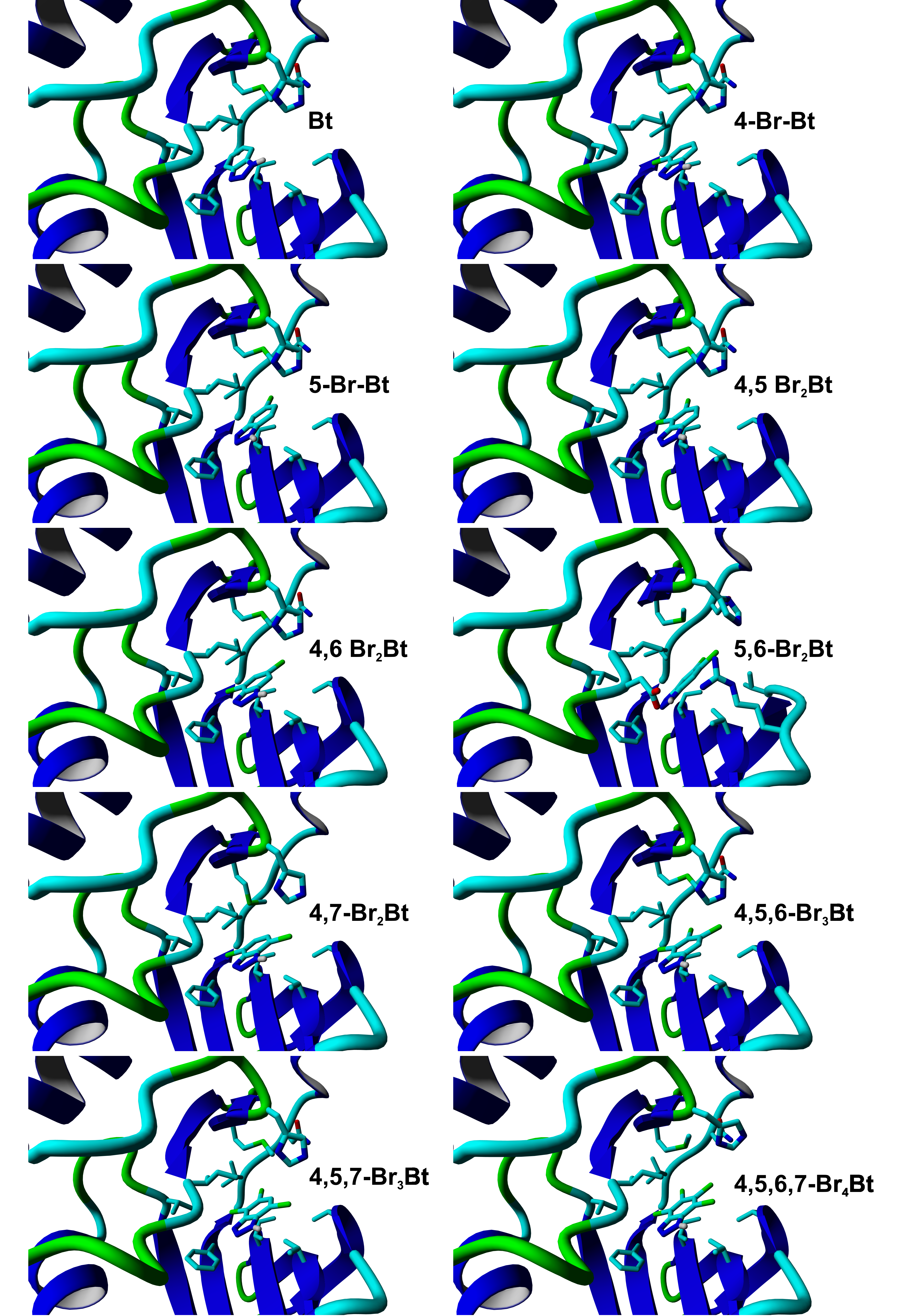

Supplement: Figure S3 — Lowest energy structures of benzotriazole and its Brominated derivatives in complex with human CK2α. (TIF) [file pone.0048898.s003.tif]
