# Supplementary material for: Isomeric Mono-, Di-, and Tri-Bromobenzo-1H-Triazoles as Inhibitors of Human Protein Kinase CK2α
Source: PLoS One. 2012 Nov 14;7(11):e48898. doi: 10.1371/journal.pone.0048898 (PMC3498355; doi:10.1371/journal.pone.0048898)
Supplement: Table S1 — Short contact between halogen atom and potential halogen bond acceptors identified in 18 of 21 complexes of CK2α with halogenated ligands, accessible in the Protein Data Bank. (DOC) [file pone.0048898.s004.doc]

**Table S1**. Short contact between halogen atom and potential halogen bond acceptors identified in 18 of 21 complexes of CK2α with halogenated ligands, accessible in the Protein Data Bank.

| **PDB** | **Halogen bond donor (X)** | | **Halogen bond acceptor (Ac)** | | **X…Ac [**Å**]** | **Angles** | | | Ref. |
| --- | --- | --- | --- | --- | --- | --- | --- | --- | --- |
|  |  |  |  |  |  | **X…Ac-C** | | **C-X…Ac** |  |
| **1J91** | **TBS**  **TBS**  **TBS**  **TBS**  **TBS** | **Br13**  **Br13**  **Br13**  **Br10**  **Br13** | **Arg47**  **Arg47**  **Arg47**  **wat339**  **wat391** | **NE**  **NH2**  **O**  **O**  **O** | **2.99**  **3.53**  **3.30**  **3.43**  **3.24** | **100**  **67**  **106** | | **165**  **133**  **164**  **96**  **137** | **[1]** |
| **1ZOE** | **K25**  **K25**  **K25**  **K25**  **K25**  **K25** | **Br1**  **Br9**  **Br10**  **Br10**  **Br10**  **Br11** | **Glu114**  **Val116**  **wat1018**  **wat1048**  **wat1209**  **wat1344** | **O**  **O**  **O**  **O**  **O**  **O** | **3.43**  **3.24**  **3.37**  **3.41**  **3.39**  **2.87** | **150**  **129** | | **139**  **176**  **99**  **149**  **145**  **126** |  |
| **1ZOG** | **K37**  **K37**  **K37**  **K37**  **K37**  **K37**  **K37**  **K37**  **K37**  **K37**  **K37**  **K37** | **Br13**  **Br11**  **Br12**  **Br10**  **Br11**  **Br10**  **Br10**  **Br10**  **Br11**  **Br12**  **Br13**  **Br13** | **Phe113**  **Glu114**  **Glu114**  **Val116**  **Val116**  **Met163**  **wat1227**  **wat1227**  **wat1227**  **wat1228**  **wat1228**  **wat1228** | **ring**  **O**  **O**  **O**  **O**  **SD**  **O**  **O**  **O**  **O**  **O**  **O** | **3.53**  **3.23**  **3.11**  **2.98**  **3.27**  **3.46**  **2.91**  **3.29**  **3.40**  **3.20**  **3.14**  **3.15** | **49**  **162**  **164**  **130**  **129**  **117** | | **154**  **144**  **144**  **175**  **173**  **80**  **125**  **103**  **111**  **115**  **106**  **116** |  |
| **1ZOH** | **K44**  **K44**  **K44**  **K44**  **K44**  **K44** | **Br12**  **Br13**  **Br10**  **Br10**  **Br10**  **Br13** | **Glu114**  **Val116**  **Asp175**  **wat1115**  **wat1157**  **wat1339** | **O**  **O**  **OD1**  **O**  **O**  **O** | **3.18**  **2.89**  **3.16**  **3.49**  **3.60**  **3.27** | **164**  **129**  **128** | | **145**  **177**  **133**  **139**  **97**  **135** |  |
| **2OXD** | **K32**  **K32**  **K32**  **K32** | **Br4**  **Br2**  **Br1**  **Br3** | **Glu114**  **Val116**  **Met163**  **wat344** | **O**  **O**  **SD**  **O** | **3.27**  **2.80**  **3.50**  **3.19** | **164**  **133**  **135** | | **147**  **176**  **66**  **138** | **[2]** |
| **2OXX** | **K22**  **K22** | **Br3**  **Br1** | **Val116**  **wat502** | **O**  **O** | **2.72**  **3.34** | **132** | | **176**  **132** |  |
| **2OXY** | **K17**  **K17**  **K17**  **K17**  **K17**  **K17**  **K17** | **Br2**  **Br2**  **Br1**  **Br1**  **Br4**  **Br3**  **Br3** | **Glu114**  **Glu114**  **Val116**  **Val116**  **wat1081**  **wat1254**  **wat1287** | **O**  **O**  **O**  **O**  **O**  **O**  **O** | **3.16**  **3.25**  **2.95**  **2.92**  **3.54**  **3.06**  **2.90** | **167**  **169**  **132**  **132** | | **149**  **142**  **176**  **174**  **155**  **118**  **126** |  |
| **2PVK** | **P45**  **P45** | **Cl27**  **Cl27** | **Asp120**  **wat526** | **OD2**  **O** | **3.38**  **3.49** | **100** | | **156**  **109** | **[3]** |
| **2QC6** | **G12**  **G12**  **G12** | **Br1**  **Br2**  **Br2** | **Lys68**  **Met163**  **wat452** | **NZ**  **SD**  **O** | **3.56**  **3.58**  **3.63** | **86**  **91** | | **97**  **84**  **150** | **[4]** |
| **3H30** | **RFZ**  **RFZ**  **RFZ** | **Cl2**  **Cl2**  **Cl2** | **Val116**  **Val116**  **Asn117** | **O**  **O**  **O** | **3.67**  **3.16**  **3.70** | **121**  **106**  **94** | | **176**  **169**  **123** | **[5]** |
| **3KXG** | **K6X**  **K6X**  **K6X**  **K6X**  **K6X**  **K6X**  **K6X**  **K6X** | **Br15**  **Br13**  **Br19**  **Br17**  **Br19**  **Br13**  **Br28**  **Br28** | **Glu114**  **Val116**  **Asp175**  **wat344**  **wat344**  **wat489**  **wat489**  **wat547** | **O**  **O**  **OD1**  **O**  **O**  **O**  **O**  **O** | **3.57**  **2.86**  **3.09**  **2.94**  **3.37**  **3.29**  **3.14**  **3.12** | **164**  **131**  **125** | | **144**  **168**  **139**  **120**  **108**  **126**  **124**  **146** | **[6]** |
| **3KXH** | **K66**  **K66**  **K66**  **K66** | **Br13**  **Br15**  **Br17**  **Br19** | **Phe113**  **Glu114**  **Val116**  **Met163** | **ring**  **O**  **O**  **SD** | **3.53**  **3.30**  **3.32**  **3.33** | **147**  **170**  **132**  **125** | | **165**  **155**  **172**  **86** |  |
| **3KXM** | **K74**  **K74**  **K74**  **K74**  **K74**  **K74** | **Br36**  **Br38**  **Br38**  **Br38**  **Br32**  **Br34** | **Glu114**  **Val116**  **Met163**  **wat422**  **wat507**  **wat507** | **O**  **O**  **SD**  **O**  **O**  **O** | **3.55**  **3.00**  **3.42**  **3.43**  **3.18**  **3.02** | **161**  **137**  **90** | | **142**  **160**  **74**  **131**  **110**  **117** |  |
| **3KXN** | **K8X**  **K8X**  **K8X**  **K8X**  **K8X**  **K8X**  **K8X**  **K8X**  **K8X** | **I19**  **I15**  **I17**  **I15**  **I19**  **I19**  **I13**  **I15**  **I17** | **Val45**  **Phe113**  **Glu114**  **Val116**  **Val116**  **wat472**  **wat479**  **wat479**  **wat480** | **O**  **ring**  **O**  **O**  **O**  **O**  **O**  **O**  **O** | **3.69**  **3.50**  **3.34**  **2.94**  **3.17**  **3.69**  **3.44**  **3.12**  **2.22** | **107**  **57**  **157**  **138**  **125** | | **115**  **146**  **144**  **160**  **173**  **166**  **112**  **120**  **169** |  |
| **3PVG** | **K68**  **K68**  **K68**  **K68** | **Br15**  **Br13**  **Br19**  **Br13** | **Val116**  **Met163**  **wat439**  **wat606** | **O**  **SD**  **O**  **O** | **2.96**  **3.66**  **2.59**  **3.44** | **134**  **127** | | **158**  **78**  **121**  **157** |  |
| **3NGA** | **3NG**  **3NG** | **Cl22**  **Cl22** | **Gly46**  **Gly46** | **O**  **O** | **3.59**  **3.60** | **68**  **68** | | **118**  **118** | **[7]** |
| **3OFM** | **4B0**  **4B0** | **BrAC**  **BrAB** | **Glu115**  **Ile117** | **O**  **O** | **3.17**  **2.94** | **162**  **138** | | **160**  **169** | **[8]** |
| **3OWK** | **Not observed** | | | | | | | | **[9]** |
| **3OWL** | **Not observed** | | | | | | | |  |
| **3RPS** | **4B0**  **4B0** | **BrAD**  **BrAC** | **Lys68**  **Asn117** | **NZ**  **O** | **3.45**  **3.35** | **101**  **120** | **126**  **113** | |  |
| **3PE1** | **Not observed** | | | | | | | |  |

1. Battistutta R, M.Mazzorana, Sarno S, Kazimierczuk Z, Zanotti G , Pinna LA (2005) Inspecting the Structure-Activity Relationship of Protein Kinase CK2 Inhibitors Derived from Tetrabromo-Benzimidazole. *Chem Biol* **12**, 1211-1219,
2. Battistutta R, Mazzorana M, Cendron L, Bortolato A, Sarno S, Kazimierczuk Z, Zanotti G, Moro S, Pinna LA ( 2007) The ATP-Binding Site of Protein Kinase CK2 Holds a Positive Electrostatic Area and Conserved water Molecules. *Chem Bio Chem*  **8**, 1804-1809.
3. Nie Z, Perretta C, Erickson P, Margosiak S, Almassy R, Lu J, Averill A, Yager KM and Chu (2007) S [Structure-based design, synthesis, and study of pyrazolo[1,5-a][1,3,5]triazine derivatives as potent inhibitors of protein kinase CK2](http://www.polarispharma.com/literature/abstracts/structure_based_design_synthesis_and_study_of_pyrazolo.html) *Bioorg Med Chem Lett* **17**, 4191-4195
4. Chilin A, Battistutta R, Bortolato A, Cozza G, Zanatta S, Poletto G, Mazzorana M, Zagotto G, Uriarte E, Guiotto A, Pinna LA, Meggio F, Moro S (2008) Coumarin as attractive casein kinase 2 (CK2) inhibitor scaffold: an integrate approach to elucidate the putative binding motif and explain structure-activity relationships. J Med Chem **51**, 752-9.
5. Raaf J, Brunstein E, Issinger OG, Niefind K (2008) The CK2alpha/CK2beta interface of human protein kinase CK2 harbors a binding pocket for small molecules. *Chem Biol* **2**, 111-117.
6. Sarno S, Papinutto E, Franchin C, Bain J, Elliott M, Meggio F, Kazimierczuk Z, Orzesko A, Zanotti G, Battistutta R, Pinna LA (2010) ATP site-directed inhibitors of protein kinase CK2: an update. *Curr Topics Med Chem,* in press!!
7. Ferguson AD, Sheth PR, Basso AD, Paliwal S, Gray K, Fischmann TO, Le HV (2011) Structural basis of CX-4945 binding to human protein kinase CK2. *FEBS Lett* **585**, 104-10.
8. Bischoff N, Olsen B, Raaf J, Bretner M, Issinger OG, Niefind K (2011) [Structure of the Human Protein Kinase CK2 Catalytic Subunit CK2α' and Interaction Thermodynamics with the Regulatory Subunit CK2β.](http://www.ncbi.nlm.nih.gov/pubmed/21241709) *J Mol Biol* **407**, 1-12.
9. Prudent R, Moucadel V, Nguyen CH, Barette C, Schmidt F, Florent JC, Lafanechère L, Sautel CF, Duchemin-Pelletier E, Spreux E, Filhol O, Reiser JB, Cochet C (2010) [Antitumor activity of pyridocarbazole and benzopyridoindole derivatives that inhibit protein kinase CK2.](http://www.ncbi.nlm.nih.gov/pubmed/21118972) *Cancer Res* **70**, 9865-74
